# Reperfusão Coronariana no Infarto Agudo do Miocárdio: Tentar o Ótimo. Executar o Possível

**DOI:** 10.36660/abc.20210500

**Published:** 2021-07-15

**Authors:** Brivaldo Markman, Sandro Gonçalves de Lima

**Affiliations:** 1 Hospital das ClínicasUniversidade Federal de PernambucoRecifePEBrasilHospital das Clínicas - Universidade Federal de Pernambuco, Recife, PE – Brasil

**Keywords:** Doenças Cardiovasculares, Infarto do Miocárdio, Reperfusão Miocárdica, Epidemiologia, Intervenção Coronária Percutânea, Fibrinolíticos

A estratégia da reperfusão da artéria relacionada ao infarto teve início nos anos 70 com a administração intracoronariana de fibrinolítico.^1,2^ Seguiram-se a administração intravenosa^3^ e a trombólise mecânica da artéria “culpada”, inicialmente com angioplastia simples e, posteriormente, com o implante dos “stents”.^4^ Todos esses procedimentos tinham um objetivo comum: diminuir a área isquêmica/necrótica, preservar músculo, melhorar a função ventricular e, consequentemente, a sobrevida.

O sucesso do procedimento é dependente do tempo de início dos sintomas até o momento da intervenção escolhida, seja ela química ou percutânea. De acordo com as diversas diretrizes, tal intervalo gira em torno de 12 h, enfatizando que quanto mais precoce é feito o diagnóstico e realizada a intervenção, melhores serão os desfechos clínicos.^5^

Estudos comparando trombólise mecânica x química têm relatado a superioridade da primeira na redução da mortalidade e complicações hemorrágicas graves, o que levou a decisão generalizada ser a estratégia principal para tratar os pacientes.^6^ Entretanto, nem todos os hospitais dispõem de laboratório de hemodinâmica e, dentre estes, uma menor quantidade apresenta estrutura para mantê-lo aberto 24 h, sete dias por semana. Estudos realizados no exterior demonstraram a factibilidade da transferência de pacientes para hospitais com possibilidade de realizar a intervenção coronariana percutânea (ICP) no infarto agudo do miocárdio (IAM)^7^ dentro do intervalo de tempo preconizado pelas melhores evidências. Quando esse tempo não pode ser alcançado, recomenda-se fibrinólise em até 30 min da chegada à primeira instituição de saúde ou fibrinólise pré-hospitalar.^8-11^

Neste estudo, de grande importância para a saúde pública e para a gestão em cardiologia, os autores destacam que em Sergipe a distribuição dos serviços que realizam ICP não é regionalizada e dentre aqueles que o fazem, apenas um, localizado em Aracaju, atende pacientes do SUS, através da regulação a partir de outras unidades de saúde, e ressaltam a importância do tempo do hospital sem ICP ao hospital com ICP como sendo o de maior impacto no tempo total.^12^ Tratam-se de retardos que ocorrem inclusive na região de Aracaju, cujos tempos, teoricamente, deveriam ser bem mais curtos do que a mediana observada de 9 h do início dos sintomas até a chegada ao hospital com ICP.

Como ressaltado pelos autores, a característica deste hospital não ser “porta aberta” justifica a proposição para os gestores em saúde reavaliarem o fluxo de atendimento ao paciente infartado no estado, inclusive elevando os percentuais de trombólise química frente à impossibilidade de transferência para centros com ICP dentro do tempo preconizado, possibilitando, assim, a redução das altas taxas de mortalidade observadas. Esses dados apresentam um impacto ainda maior quando verificamos que quase 15% dos pacientes passaram por duas unidades hospitalares antes de chegarem ao destino final.^12^ A análise pormenorizada do subgrupo de pacientes (1,7%) que tiveram acesso direto ao hospital com ICP pode fornecer dados mais consistentes com a necessidade de mudança neste fluxo de atendimento.

A região de saúde Glória é uma das mais distantes da capital, o que pode explicar os tempos mais prolongados do hospital sem ICP ao hospital com ICP e do início dos sintomas até a chegada a esse hospital. Esses dados justificam o mais baixo percentual de ICP primária (17,1%) dentre as regiões do estado. Apesar disso, o estudo mostra a menor taxa de mortalidade (7,5%) nessa mesma região. É importante ressaltar o menor percentual de diabéticos, a menor média de idade e a mais alta taxa de realização de ICP não primária nessa região. Em contrapartida, a região de Estância, cuja taxa de mortalidade foi a mais alta (18,6%), é vizinha da região de Aracaju, o que justifica um tempo do hospital sem ICP ao hospital com ICP quase 5 horas menor, e do início dos sintomas até a chegada ao hospital com ICP 5 h e 30 min menor, e, dessa forma, explica um percentual mais elevado de ICP primária (46,6%). Entretanto, essa região apresenta a maior média de idade e a segunda taxa mais elevada de diabetes. A avaliação global desses dados nos permite levantar a hipótese de que o tempo para o atendimento ao quadro agudo possa ter sido compensado pelo perfil de risco heterogêneo característico da população estudada, o que justifica a não diferença nas taxas de mortalidade nos dados ajustados.

O atendimento ao paciente infartado continua sendo um desafio para cardiologistas e gestores em saúde, especialmente em localidades remotas e com recursos escassos. É possível que nos últimos meses a pandemia de COVID-19 tenha acentuado essas desigualdades, mas enquanto, guiados pela ciência, buscarmos o ótimo, devemos persistir na luta pela execução de medidas possíveis: identificação das causas dos maiores retardos no atendimento, distribuição geograficamente igualitária e simétrica de centros de atendimento em cardiologia, estabelecimento de diretrizes para ampliação do uso da trombólise química, incluindo a trombólise pré-hospitalar, que contribuirão para reduzir as taxas de mortalidade de Propriá a Estância e de Glória a Aracaju.^12^
