# Supplementary material for: Reperfusão Coronariana no Infarto Agudo do Miocárdio: Tentar o Ótimo. Executar o Possível
Source: Arq Bras Cardiol. 2021 Jul 15;117(1):130–1. [Article in Portuguese] doi: 10.36660/abc.20210500 (PMC8294741; doi:10.36660/abc.20210500)
Supplement: Translation

# Translation: Coronary Reperfusion in Acute Myocardial Infarction: Trying the Optimal. Executing the Possible

**Authors:** Brivaldo Markman, Sandro Gonçalves de Lima

**Affiliations:** 1 BrasilHospital das Clínicas - Universidade Federal de Pernambuco, Recife, PE – Brasil

**Keywords:** Cardiovascular Diseases, Myocardial Infarction, Myocardial Reperfusion, Percutaneous Coronary Intervention, Fibrinolytic Agents

The infarction-related artery reperfusion strategy began in the 1970s with the intracoronary administration of fibrinolytics.^1,2^ This was followed by intravenous administration^3^ and mechanical thrombolysis of the “culprit” artery, initially with simple angioplasty and, later, with stenting.^4^ All of these procedures had a common goal: to reduce the ischemic/necrotic area, preserve muscle, improve ventricular function and, consequently, survival.

The success of the procedure depends on the time from the onset of symptoms to the moment of the chosen intervention, whether chemical or percutaneous. According to different guidelines, this interval is about 12 hours. The earlier the diagnosis and intervention, the better the clinical outcomes will be.^5^

Studies comparing mechanical x chemical thrombolysis have reported the superiority of the former in reducing mortality and severe hemorrhagic complications, which led the general decision to be the main strategy to treat patients.^6^ However, not all hospitals have a cath lab and, among them, a smaller number has the structure to keep it open 24 hours, 7 days a week. Studies conducted abroad have demonstrated the feasibility of transferring patients to hospitals with the possibility of performing percutaneous coronary intervention (PCI) in acute myocardial infarction (AMI)^7^ within the time interval recommended by the best evidence. When this time interval cannot be fulfilled, fibrinolysis within 30 minutes of arrival at the first health institution or pre-hospital fibrinolysis is recommended.^8-11^

In this study, which is of great importance for public health and cardiology management, the authors point out that in Sergipe the distribution of services that perform PCI is not regionalized and among those that do it, only one, located in Aracaju, serves patients referred from the public health system, through regulation from other health units, and emphasize the importance of time from the hospital without PCI to the hospital with PCI as having the greatest impact on the total time.^12^ These are delays that occur even in Aracaju, whose times, theoretically, should be much shorter than the observed median of 9 h from the onset of symptoms to arrival at the hospital with PCI.

As pointed out by the authors, the fact that this hospital does not have “open doors” justifies the proposition for health managers to reassess the flow of care for infarcted patients in the state, by increasing the percentages of chemical thrombolysis due to the impossibility of transferring them to centers with PCI within the recommended time, thus making it possible to reduce the high mortality rates observed. These data have an even greater impact if we consider that nearly 15% of patients went through two hospital units before reaching their final destination.^12^ A detailed analysis of the subgroup of patients (1.7%) that had direct access to the hospital with PCI can provide data that are more consistent with the need to change this flow of care.

The Glória health region is one of the most distant from the capital, which may explain the longer times from the hospital without PCI to the hospital with PCI and from the onset of symptoms to arrival at that hospital. These data justify the lowest percentage of primary PCI (17.1%) among the regions of the state. Despite this, the study shows the lowest mortality rate (7.5%) in this region. It is important to highlight the lowest percentage of diabetics, the lowest average age and the highest rate of non-primary PCI in this region. On the other hand, the region of Estância, the mortality rate of which was the highest (18.6%), is close to the region of Aracaju, which justifies a time almost 5 hours shorter from the hospital without PCI to the hospital with PCI, and from onset of symptoms to arrival at the hospital with PCI 5 h and 30 min shorter, which explains a higher percentage of primary PCI (46.6%). However, this region has the highest average age and the second highest rate of diabetes. The global assessment of these data allows us to hypothesize that the time to care for the acute condition may have been compensated by the heterogeneous risk profile of the study population, which justifies the non-difference in mortality rates in the adjusted data.

Care of infarcted patients remains a challenge for cardiologists and health managers, especially in remote locations with scarce resources. It is possible that in recent months the COVID-19 pandemic has accentuated these inequalities, but while, guided by science, we seek the optimal, we must persist in the fight for the implementation of possible measures: identification of the causes of the greatest delays in care, geographically equal and symmetrical distribution of cardiology care centers, establishment of guidelines for expanding the use of chemical thrombolysis, including pre-hospital thrombolysis, which will contribute to reducing mortality rates from Propriá to Estância and from Glória to Aracaju.^12^
